# The evaluation of cEEGrids for fatigue detection in aviation

**DOI:** 10.1093/sleepadvances/zpae009

**Published:** 2024-02-06

**Authors:** Carmen van Klaren, Anneloes Maij, Laurie Marsman, Alwin van Drongelen

**Affiliations:** Royal Netherlands Aerospace Centre (NLR), Department of Safety and Human Performance, Amsterdam, The Netherlands; Royal Netherlands Aerospace Centre (NLR), Department of Safety and Human Performance, Amsterdam, The Netherlands; Royal Netherlands Aerospace Centre (NLR), Department of Safety and Human Performance, Amsterdam, The Netherlands; Royal Netherlands Aerospace Centre (NLR), Department of Safety and Human Performance, Amsterdam, The Netherlands

**Keywords:** EEG analysis, sleep deprivation, sleepiness

## Abstract

Operator fatigue poses a major concern in safety-critical industries such as aviation, potentially increasing the chances of errors and accidents. To better understand this risk, there is a need for noninvasive objective measures of fatigue. This study aimed to evaluate the performance of cEEGrids, a type of ear-EEG, for fatigue detection by analyzing the alpha and theta power before and after sleep restriction in four sessions on two separate days, employing a within-participants design. Results were compared to traditional, highly validated methods: the Karolinska Sleepiness Scale (KSS) and Psychomotor Vigilance Task (PVT). After sleep restriction and an office workday, 12 participants showed increased alpha band power in multiple electrode channels, but no channels correlated with KSS scores and PVT response speed. These findings indicate that cEEGrids can detect differences in alpha power following mild sleep loss. However, it should be noted that this capability was limited to specific channels, and no difference in theta power was observed. The study shows the potential and limitations of ear-EEG for fatigue detection as a less invasive alternative to cap-EEG. Further design and electrode configuration adjustments are necessary before ear-EEG can be implemented for fatigue detection in the field.

**Statement of Significance**

The cEEGrid device implements the neurophysiological method of electroencephalography (EEG) through an array with ten gel electrodes that are fitted around the ear. In this study, the performance of cEEGrids was evaluated for fatigue detection against traditional fatigue detection methods—the Karolinska Sleepiness Scale and the psychomotor vigilance task—in a controlled experimental setting. The results showed its promise in detecting mild sleep loss-related alpha power changes. The findings of this research lay the groundwork for future studies, showing not only the potentials, but also suggesting improvements for effective application of the cEEGrids as a fatigue detection tool in the field.

## Introduction

According to the International Civil Aviation Organization [1], fatigue is defined as a physiological state of reduced mental or physical performance capability resulting from sleep loss or extended wakefulness, circadian phase, or workload (mental and/or physical activity) that can impair a crew member’s alertness and ability to safely operate an aircraft or perform safety-related duties. The causes of fatigue may be attributed to multiple factors, such as increased sleep debt, disturbed circadian rhythm, and poor sleep quality [2]. Fatigue leads to reduced activity in various areas of the brain, such as the frontal, temporal, and parietal regions, thereby impairing performance on tasks that require vigilance and visuospatial attention [3, 4]. Consequently, the risk of human errors increases, which is problematic in safety-critical sectors, where errors can lead to fatal accidents [5, 6]. For instance, pilots and air traffic controllers need to maintain a high level of alertness to accurately process and respond to changes [7, 8]. The aviation industry operates 24 hours per day with duty periods often occurring at night. This can lead to circadian disruption and sleep restriction that contribute to a disturbed sleep–wake rhythm, increasing the probability of high fatigue levels [3, 9, 10]. For instance, according to a study among commercial Portuguese airline pilots, 90% of pilots experience severe fatigue, while 59% report significant daytime sleepiness [11]. Moreover, it is estimated that human fatigue is a contributing factor to 15%–20% of all aircraft accidents [12].

Currently, there are various methods for determining fatigue or sleepiness, including subjective measures that rely on self-evaluation, such as the nine-point Karolinska Sleepiness Scale (KSS) [13], and objective measures that consider performance and physiological factors [14], such as the psychomotor vigilance task (PVT). Subjective methods do not always accurately reflect performance, as studies have shown that individuals may be unaware of the decline in their cognitive performance and may therefore inadvertently provide false estimations [14–16]. Consequently, such inaccurate estimations pose a potential risk to flight safety when operators are on duty [17]. For this reason, the KSS is often combined with objective measures, such as the PVT, which measures sustained attention based on a simple reaction time task. Sustained attention or vigilance is defined as the ability to maintain attention and to react to changes at random intervals over an extended period of time [18, 19]. An increase in fatigue and sleepiness is associated with a decrease in response speed of the vigilance performance measure [14, 20]. Both the KSS and PVT have been demonstrated to be reliable and sensitive measures of fatigue, but since this combination cannot be carried out simultaneously with another activity, they require an amount of engagement of the participant that is not always feasible during flight-related operations. Likewise, these methods only capture one instance, so they are not suitable for continuous monitoring [5]. To conduct more comprehensive research on fatigue in aviation as well as explore the possibility of real-time alerting systems, it is necessary to be able to continuously monitor the fatigue status of operators in a noninvasive and objective manner.

Neurophysiological method of electroencephalography (EEG) is a viable and reliable objective measure of fatigue [3, 5], that can be applied in real time. EEG measures electrical activity through electrodes that are placed on the scalp. Electrical activity is measured in different frequency bands, such as delta (2–4 Hz), theta (4–8 Hz), alpha (8–12 Hz), and beta (13–32 Hz) waves [3]. Increases in alpha and theta activity are related to mental fatigue, sleepiness, and decreased alertness [14, 19, 21–23]. For instance, Gorgoni et al. [24] and Kaida et al. [25] showed significant correlations between PVT performance, KSS scores, and EEG alpha and theta activity after sleep deprivation and in normal sleep–wake cycles. Additionally, Huang et al. [26] demonstrated the feasibility of EEG as a real-time fatigue detection and alerting system by examining alpha and theta power. However, even though EEG is frequently used to detect fatigue, the conventional setup is unsuitable for use in flight-related operations due to, for instance, the lengthy preparation time and limited mobility of the attached cap and electrodes] [3, 14, 27–29]. Nevertheless, the technology is still evolving, and manufacturers are increasingly developing innovative solutions to make EEG less invasive and more mobile [3, 5, 28, 30]. Devices are under development that integrate EEG in headphones placed around or inside the ear for use outside of laboratory settings, such as the Neurable Enten headphones [31] or the Emotiv Epoc headset [32]. This is a promising development for the aviation domain, considering the use of headsets among operators in this field with the benefit of unobstructed view [28, 33–35].

A similar, but somewhat more intrusive development is the cEEGrid device, a thin array with ten gel electrodes that are fitted around the ear. The cEEGrids hardly disturb normal movement, because the electrodes can be connected to a micro wireless amplifier, and worn for an extended duration of at least seven hours [33]. The performance of cEEGrids in capturing event-related potentials and differences in EEG oscillations has already been validated through comparisons with cap-EEG [33, 36–38]. For instance, temporal alpha power variations between open and closed eyes were demonstrated in approximately 40% of the electrodes [39]. Additionally, studies have shown promising results by differentiating sleep stages, levels of mental workload, and decreases in auditory attention using cEEGrids [3, 5, 33, 38]. However, as of the time of writing, we are not aware of any studies that reported to have evaluated the performance of cEEGrids for fatigue detection. Before EEG devices around the ear can be used in the field, their performance in measuring fatigue must be studied by comparing outcomes with other fatigue measures within a controlled experimental setting.

Therefore, the aim of this study is to evaluate the performance of cEEGrids as an objective measure for fatigue detection. To achieve this, the relationship between EEG data from separate cEEGrids electrodes and the outcomes of the traditional fatigue detection methods in aviation, PVT and KSS, is studied. Additionally, this research investigates the effects of different times of day (morning versus afternoon) and conditions (control vs. sleep restriction) on these methods. It is expected that sleep restriction, achieved by waking up three hours earlier than usual, and longer time awake in the afternoon will result in increased subjective sleepiness and a decrease in vigilance performance. Furthermore, the relevant EEG results measured by the cEEGrids electrodes are expected to show an increase in relative alpha and theta band power due to sleep restriction with varying magnitude across channels [8, 39, 40]. Therefore, the first hypothesis is that the relative alpha and theta band power of channels and KSS scores are higher, and that task performance on the PVT is lower after sleep restriction due to an increase in fatigue. Second, following an 8-hour workday, identical outcomes in EEG band power, KSS scores, and PVT performance are expected after an increase in fatigue in the afternoon compared to the morning and it is expected that this increase would be greater after sleep restriction [41, 42]. Finally, comparing cEEGrids to traditional means, it is hypothesized that relative alpha and theta band power negatively correlate with task performance of the PVT and positively correlate with self-reported KSS scores.

## Materials and Methods

### Participants

Fifteen participants were recruited, all employees from the Royal Netherlands Aerospace Center (NLR). None of the participants reported taking any medication that could affect sleep or having neurologic diseases or sleep disorders (e.g. insomnia and sleep apnea). Additionally, all participants reported having a regular sleep schedule, sleeping between 6 and 8 hours per night, having normal or corrected-to-normal vision, and were willing to sign an informed consent form. They received compensation for their participation in the form of lunch coupons. One participant was unable to restrict hours of sleep during the night, and was therefore excluded from further analyses examining effects of sleep restriction. A priori power analysis was performed for a 2 × 2 within-participant repeated-measures ANOVA to calculate the sample size using G*Power 3.1.9.7 [43]. The analysis was based on a large effect size (Cohen’s *f* = 0.40), an alpha level of 0.05, and a desired power of 0.80. The analysis indicated that a sample size of 12 participants would be required.

### Design

For this study, a 2 × 2 within-participant, repeated-measures design was conducted. The experiment was divided into 2 days with either no sleep restriction (control) or with sleep restriction of 3 hours as within-participants factor. Participant fatigue was measured in both a morning and an identical afternoon session, with “time of day” as the second within-participant factor, in order to compare fatigue levels and experiment results at the start and following an 8-hour workday. In total, four measurements were taken over the course of 2 days of participation with 1 week apart. The study was approved by the Ethics Review Board of the Faculty of Social and Behavioral Sciences of Utrecht University.

### Questionnaires

The questionnaires inquired about demographic information, including gender, age, and handedness. Additionally, participants provided information on at what time they went to bed, their sleep quality, and their time of awakening. They also completed the KSS. The KSS ranges from 1 (very alert) to 9 (very sleepy, fighting sleep). Previous research has indicated that a KSS score greater than seven is a validated indicator of fatigue, implying a need for rest and potentially risking performance and safety in various settings [44, 45]. Sleep quality of the preceding night was assessed using the Groningen Sleep Quality Scale (GSQS). The GSQS utilized an anchored scoring system ranging from 0 to 14, where a higher score indicates a lower perceived quality of sleep, and a score of 0 to 2 points indicates unrestricted and undisturbed sleep. A score above seven serves as an indicator of disturbed sleep [46].

### NLR Study app

The NLR Study app is a smartphone application to collect data for internal non-health-related NLR studies. In this study, the app was used to present the questionnaires to participants on their own mobile devices [47].

### Psychomotor vigilance task

**Figure 1.**
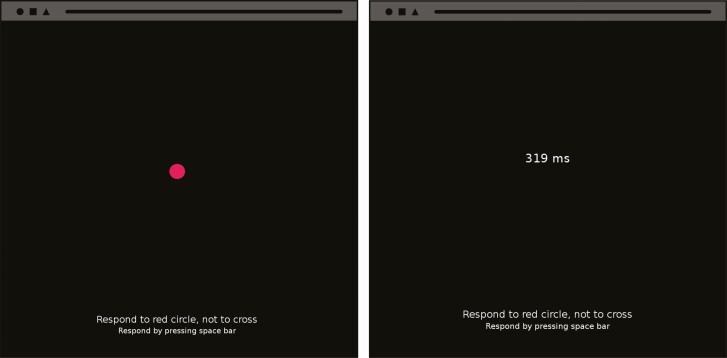
Psychomotor vigilance task. Stimulus (left), feedback reaction time (right).

The 10-minute PVT is a reaction test and was used to assess sustained attention (Figure 1). A decrease in PVT performance, characterized by longer reaction times, has been significantly related to increased subjective and objective fatigue among pilots [5, 18, 20, 48]. The PVT was administered on a standard laptop using the open-source psychology experiment building language framework [49]. During the task, participants were instructed to press the spacebar as quickly as possible in response to a red circle presented on the screen at random intervals between 2 and 10 seconds. To account for variations in participants’ average reaction times, the common threshold of > 500 ms for lapses was deemed too strict [50]. Therefore, individualized thresholds were calculated instead, using a 95% confidence interval based on two standard deviations [51]. Responses falling within the interval were considered valid, while responses outside the interval were classified as false starts (<5%) or lapses (>95%). These responses were subsequently excluded from the final analyses. Following each response, feedback on the reaction time was displayed to the participant for a duration of 1000 ms. The task consisted of 75 trials in total. Task performance was assessed by computing the mean response speed (1/RT*1000) [19, 20, 50].

### cEEGrids

**Figure 2.**
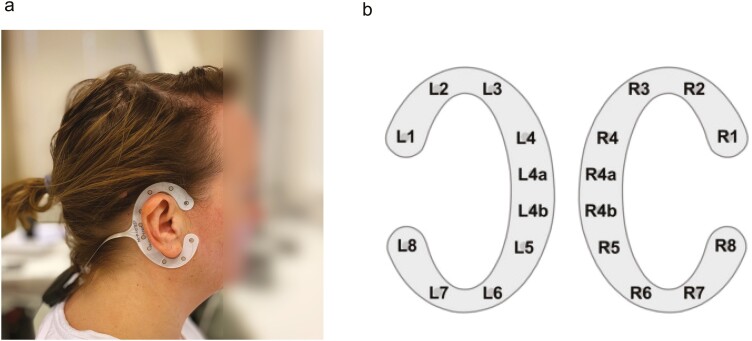
Experimental setup cEEGrid electrodes. *Note. (A*) Position cEEGrid array behind ear (B) Channel layout of left and right ear [52].

EEG data were recorded using two cEEGrid devices (TMSi, Oldenzaal, Netherlands). Each device consisted of ten Ag/AgCl electrodes attached around one of the participant’s ears using double-sided adhesive (Figure 2) [33]. Additionally, two ocular electrodes (VEOG) were placed above and below the left eye to record vertical eye movements. Furthermore, electrode channel R4b, located directly behind the right ear, served as a reference electrode, while an electrode on the wrist was used as the ground. The cEEGrid electrodes were transmitted to the SAGA TMSi amplifier (TMSi, the Netherlands) with a sampling rate of 500 Hz. The recorded data were saved using Polybench 1.34 (TMSi, the Netherlands). At the start of recording, the impedance of most electrodes was maintained below 30–50 kOhm. However, an impedance range of 50–100 kOhm for a maximum of three electrodes is deemed acceptable, adhering to the recommendation to minimize interference after application [36].

### Procedure

**Figure 3.**
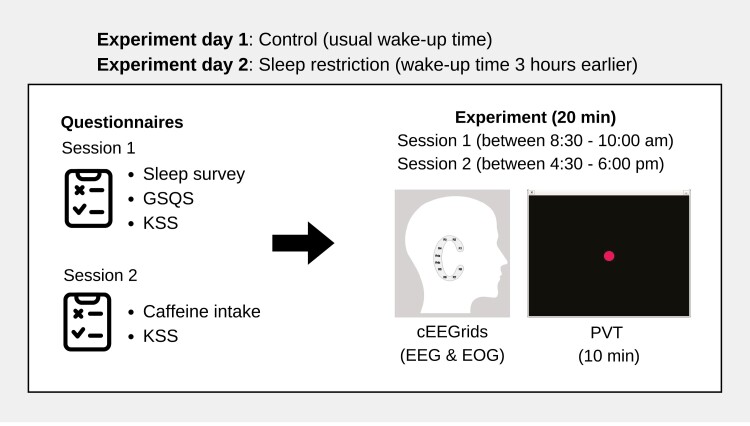
Experimental procedure. Timeline of the procedure of both test days starts with answering surveys about amount of sleep, GSQS, and the KSS. The 20-minute morning session starts between 8:30 and 10:00 am and the afternoon session between 4:30 and 6:00 pm. In all sessions, a 10-minute psychomotor vigilance task (PVT) is conducted while EEG is measured using the cEEGrid devices. At the start of the afternoon session on both days, surveys on caffeine intake and KSS are answered.

The study was conducted over two separate days separated by 1 week wash-out period (Figure 3). Prior to the start of the experiment, participants received a short briefing about the study and were asked to complete the questionnaire about their demographics on the NLR Study app and to give their informed consent. On the first day, participants were instructed to wake up at their usual time. They participated in two different sessions of 20 minutes with one session in the morning between 8:30 and 10:00 am and one in the afternoon between 4:30 and 6:00 pm. The second condition took place 1 week later with added sleep restriction. Participants were instructed to wake up 3 hours earlier than their usual wake-up time. Condition order was not randomized. Participants continued with their regular workday activities in between sessions. They were instructed to avoid caffeine intake 30 minutes before each session, and alcohol and drugs for 24 hours prior to the experiment day.

The morning session began with participants filling out a survey on the NLR Study app with sleep-related questions. Additionally, at the beginning of the afternoon session, participants were requested to fill out a survey with questions on caffeine intake during the experiment day. For all sessions, participants answered the KSS while the cEEGrids were applied around their ears, and VEOG electrodes above and below the left eye by the experimenter. Subsequently, participants performed the PVT for 10 minutes with open eyes while EEG data were recorded. Measurements took place in a testing room with controlled lighting. The experimenter remained outside the participant’s central field of vision. At the end of the second experiment day, participants who felt too fatigued to safely drive home were provided with the option of resting in a bed or receiving compensation to travel home using public transport.

### Data analysis

The cEEGrid data were processed with EEGLAB (version 2020.0; Delorme and Makeig, 2004) using the cEEGrid EEGLAB plugin (version 0.9) in MATLAB (The MathWorks, Natik, USA)(v.2022a) [52, 53]. Data were down-sampled to 250 Hz and band-pass filtered with a high-pass filter at 1 Hz and low-pass filter at 40 Hz. Furthermore, data were offline re-referenced to algebraically linked mastoids (channels L4b and R4b) and reference channels were removed, leaving 9 electrodes per ear. Following Hölle and Bleichner [36] Artifact Subspace Reconstruction was implemented using the clean raw data plugin (ver.2.3) for EEGlab (flatline criterion = 60, high-pass = [0.25 0.75], channel criterion = off, line noise criterion = off, burst criterion = 20, and window criterion = off). independent component analysis was run to correct for remaining artifacts. Components were visually inspected and removed. For spectral analysis, power spectral density was computed using Welch’s method in epochs of 2 seconds with 50% overlap. The relative power in the theta (4–7 Hz) and alpha (8–12 Hz) bands was calculated for each channel by


$$\begin{array}{l} RP=\frac{P(x)}{P(sum)}*100\% \end{array}$$

where P(x) indicates the band power, and P(sum) the total band power from 1 to 40 Hz. Relative band power was averaged for each channel [54].

### Statistical analyses

Statistical analyses were performed with IBM SPSS Statistics (Version 20). Paired samples *t*-tests were conducted to test whether there was a significant decrease in both hours of sleep and scores of the GSQS on the day following sleep restriction. Furthermore, KSS scores and mean response speed of PVT were analyzed by a two-way repeated-measures ANOVA with condition (control vs. sleep restriction) and ToD (morning vs. afternoon) to study whether there was a significant increase in KSS scores and decrease in PVT performance after a workday and sleep restriction. Furthermore, a 2 × 2 × 18 repeated-measures ANOVA was conducted, incorporating within-participant factors for ToD (morning vs. afternoon), condition (control vs. sleep restriction), and electrode channels. This was conducted to determine whether there was a significant increase in relative alpha and theta band power, as measured by cEEGrids, following a workday and sleep restriction, and to control for any differences across the electrode channels. Finally, to test the third hypothesis comparing fatigue detection by traditional means (KSS and PVT) and fatigue detection by cEEGrids band power, repeated-measures correlations (rmcorr) between KSS scores, PVT performance, and relative alpha and theta band power were conducted for collective results [55]. Statistical analysis was performed using an alpha of *p* < 0.05 for all tests. Results were corrected for multiple comparisons using the Benjamini–Hochberg procedure (*q* = 0.1) when deemed necessary [56].

## Results

### Behavioral data

The final sample consisted of fourteen participants (5 female, 9 male; 3 left-, 11 right-handed) aged between 25 and 58 years (*M* = 34.93, *SD* = 10.63). Descriptive statistics of the demographics and experiment outcomes are presented for each condition and ToD in Table 1. To verify whether sleep restriction of participants had occurred, a paired samples *t*-test was conducted on the amount and quality of sleep in both conditions. Participants indeed slept significantly longer on the control condition than after sleep restriction, *t*(14) = 6.99, *p* < .01. Moreover, quality of sleep was rated better in the control condition compared to the sleep-restricted day, *t*(14) = 3.29, *p* < .01. Furthermore, a paired samples *t*-test was conducted for caffeine intake. There was an increase in caffeine intake on the sleep restriction day, *t*(14) = −2.88, *p* = 0.013.

**Table 1.** Means and Standard Deviations of Behavioral Data, KSS, and PVT (*N* = 14)

Condition	Control				Sleep restriction			
	*M*		*SD*		*M*		SD	
Time of awakening	6:54 am		135.90 (min)		4:19 am		86.90 (min)	
Bedtime	11:29 pm		62.30 (min)		11:17 pm		60.0 (min)	
Hours of sleep	7.12		0.70		4.94		1.07	
GSQS	3.21		2.67		7.43		4.20	
Caffeine (mg)	217.14		122.56		257.14		142.40	

Time of day	Morning		Afternoon		Morning		Afternoon	
	*M*	*SD*	*M*	*SD*	*M*	*SD*	*M*	*SD*
Hours of time awake	2.14	0.71	10.0	0.57	4.43	0.74	12.43	0.74

Groningen Sleep Quality Scale (GSQS), caffeine intake was based on amount of coffee cups (average of 80 mg a cup).

**Figure 4.**
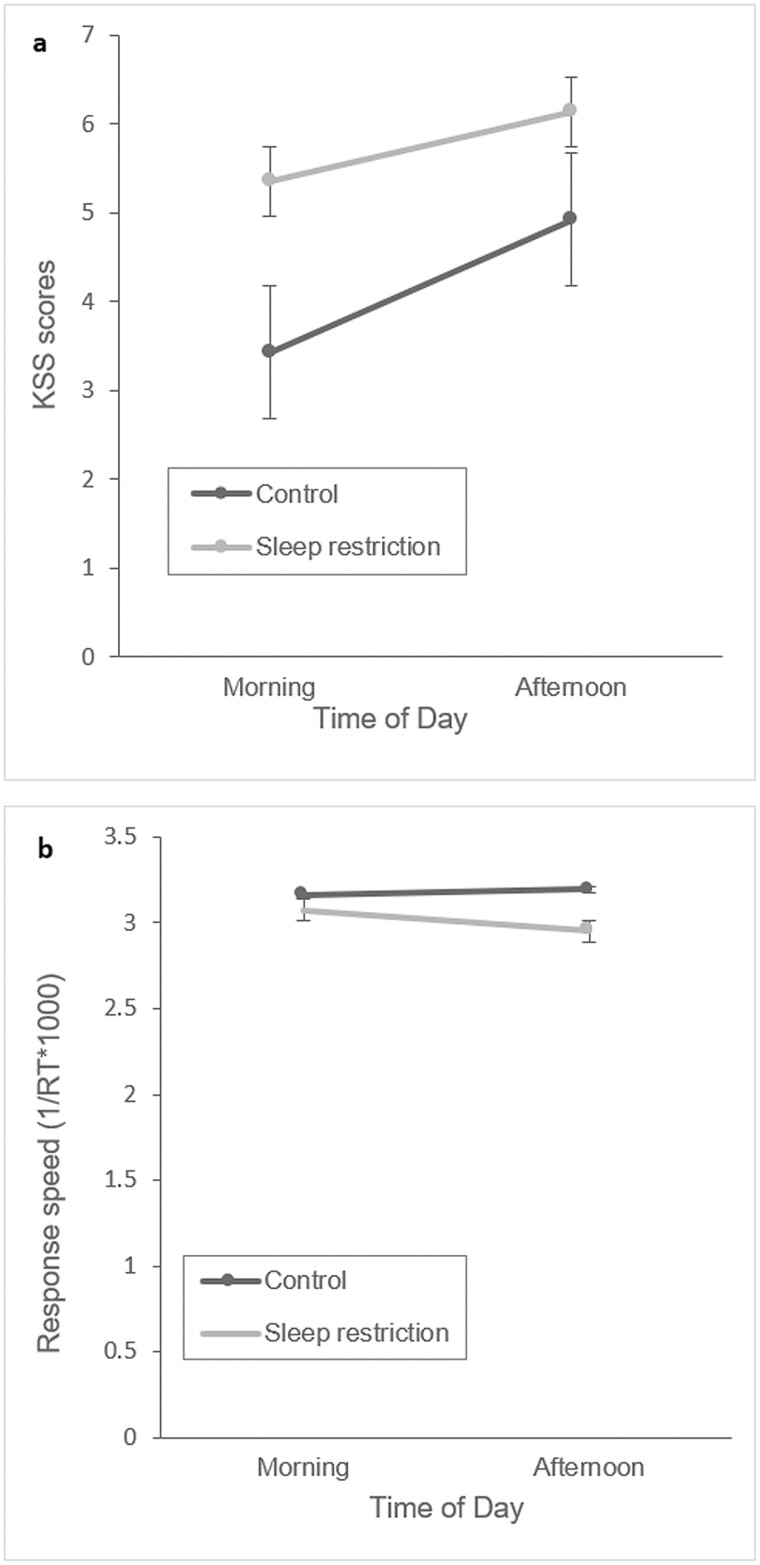
Changes in mean KSS scores and response speed of the PVT as a function of condition (control and sleep restriction) and time of day (morning and afternoon). (A) Mean Karolinska Sleepiness Scale (KSS) scores in the morning and afternoon divided by within-participant factor condition (control and sleep restriction). (B) Mean response speed (1/RT*1000) of the Psychomotor Vigilance Task (PVT) in the morning and afternoon divided by within-subject factor condition (control and sleep restriction). Note that a high response speed indicates better PVT performance.

A two-way repeated-measures ANOVA was conducted to test the first and second hypotheses on subjective fatigue detection method, KSS, by examining the effect of condition (control vs sleep restriction) and tod (morning vs. afternoon) on KSS scores (Table 1). KSS scores were significantly higher after sleep restriction, thus the within-participants factor condition had a significant effect on the KSS scores, F(1,13) = 6.87, *p* = .021, ηp² = 0.346. Second, ToD had a significant and strong effect on KSS scores with higher scores in the afternoon, F(1,13) = 10.47, *p* < .01, ηp² = 0.446. However, the interaction between ToD and condition was found not to be significant, F(1,13) = 0.77, *p* = .397, ηp² = 0.056. The mean scores of the KSS are illustrated in Figure 4A.

To sum up, participants experienced mild sleep restriction (<5 hours) and a decrease in sleep quality as a result of the experimental setup. Their subjective fatigue, as rated by means of the KSS, significantly increased in the afternoon after a workday, and in both the morning and afternoon tests after sleep restriction.

### Psychomotor vigilance task

To test the first and second hypotheses for PVT performance, the effect of condition and ToD on mean response speed of the PVT were analyzed using a two-way repeated-measures ANOVA with two levels of day (control, fatigue) and two levels of ToD (morning, afternoon) as within-participants factors (Table 1). First, no statistically significant effect of condition was found for mean response speed of the PVT, F(1,13) = 4.04, *p* = .066, ηp² = 0.125. Second, the within-participants effect of ToD on the response speed was also found not to be statistically significant F(1,13) = 1.24, *p* = .286, ηp² = 0.237. However, a significant and strong interaction effect was found between ToD and condition, F(1,13) = 6.33, *p* = .026, ηp² = 0.328, with a difference of mean response speed in condition depending on factor ToD, as illustrated in Figure 4B. Specifically, pairwise comparisons indicated that there was only a slower mean response speed in the afternoon of the sleep-restricted day when compared to the morning (*p* < .05). However, there were no differences found between the mornings of both conditions neither between the morning and afternoon tests on the control day. To summarize, PVT performance decreased in the afternoon of the sleep restriction condition, as demonstrated by a slower response speed.

### EEG spectral analysis

**Figure 5.**
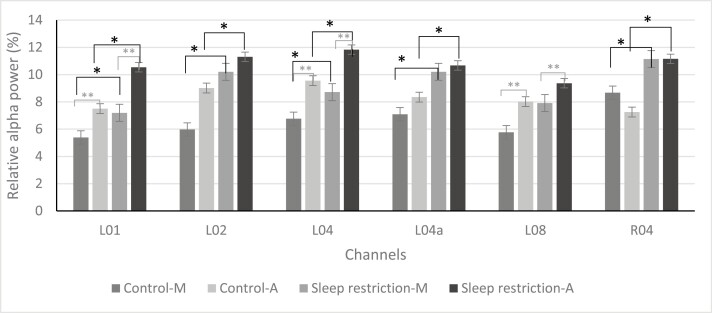
Relative alpha band power of channels L01, L02, L04, L04a, L08, and R04. Effect of time of day and condition on mean relative alpha band power (*N* = 12). *M* = morning test; A, afternoon test. *significant effect condition (*p* < 0.05), **significant effect ToD (*p* < 0.05).

A Repeated-Measures ANOVA was conducted with within-subject factors condition, ToD, and channels to test the first and second hypotheses and to verify the performance in capturing changes in alpha and theta power of all cEEGrid channels. As a number of EEG recordings were heavily artifact-laden, the final population for the EEG analysis was 12. Results showed a significant and large difference between channels in relative alpha power, F(17,1) = 5.33, *p* < .01, ηp² = 0.348. Furthermore, significant and strong interaction effects were found between condition and channel, F(17,1) = 23.41, *p* < .01, ηp² = 0.229, and ToD and channel, F(17,1) = 17.46, *p* < .01, ηp² = 0.202. As there were significant differences between channels, Bonferroni-corrected pairwise comparisons in the ANOVA model revealed a main effect of condition (*p* < .05) for channels L01, L02, L04, L04a, and R04. Furthermore, a main effect of ToD (*p* < .05) was found for channels L01, L04, and L08 with an increase in relative alpha band power in the afternoon tests (Figure 5). There was no significant interaction effect between ToD and condition.

**Figure 6.**
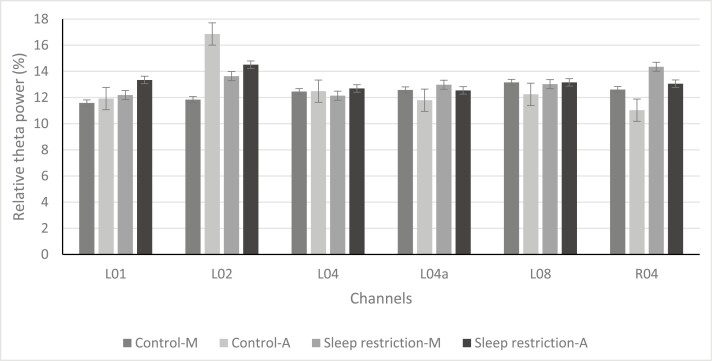
Relative Theta band power of channels L01, L02, L04, L04a, L08, and R04. Effect of time of day (ToD) and day on mean relative theta band power (*N* = 12). M, morning test; A, afternoon test.

The effect of condition, ToD, and channels on relative theta band power were analyzed in a repeated-measures ANOVA and a medium significant main effect of channel was found, F(17,1) = 2.34, *p* < .01, ηp² = 0.190. However, there were no other main or interaction effects (Figure 6).

To sum up, for 75% of channels (13 out of 18 channels), there was no increase in relative alpha band power after sleep restriction was found. For 83% of channels (15 out of 18 channels), there was no difference in relative alpha band power found after a workday either. There were no differences found in theta power for condition and ToD.

### Correlations between KSS, PVT, and relative alpha band power

To test the third hypothesis, repeated-measures correlations were performed on all measurements collectively of KSS scores, PVT response speed, and relative alpha band power of a selection of channels (L01, L02, L04, L04a, L08, and R04), as determined by follow-up tests following the ANOVA analysis. These correlations aimed to investigate the potential associations between the traditional measures, KSS and PVT, and changes in alpha band power measured by cEEGrids. Contrary to the hypotheses, results showed no significant correlations between KSS scores and relative alpha power of the channels. Moreover, there was no significant correlation between PVT response speed and relative alpha power of the channels (Table 2).

**Table 2.** Repeated-Measures Correlations Between Mean KSS, PVT Response Speed and Relative Alpha Band Power of Channels (*N* = 12)

	KSS	PVT	L01	L02	L04	L04a	L08	R04
KSS	—							
PVT	−0.395**	—						
L01	0.241	−0.026	—					
L02	0.176	−0.069	0.345^*^	—				
L04	0.048	0.005	0.628**	0.657^**^	—			
L04a	0.068	0.061	0.423^**^	0.356^*^	0.641^**^	—		
L08	0.272	0.104	0.639^**^	0.443^**^	0.542^**^	0.443^**^	—	
R04	−0.012	0.034	0.425^**^	0.241	0.391^*^	0.613^**^	0.453^**^	—

*p < .05 **p < .01. *p*-values are corrected for multiple comparisons. Karolinska Sleepiness Scale (KSS), Psychomotor Vigilance Task (PVT), response speed (1/RT *1000).

## Discussion

This study aimed to evaluate the performance of cEEGrids as an objective measure of fatigue by comparing their performance with established fatigue detection methods (KSS and PVT). The cEEGrids demonstrated an increase in relative alpha power for five channels after sleep restriction. Additionally, results showed an increase in KSS scores and a decrease in PVT performance after sleep restriction. Similar findings were observed after a workday for KSS scores and PVT response speed, along with an increase in relative alpha power for three channels. However, no significant correlations were found between KSS scores, PVT response speed, and relative alpha power.

### The effect of fatigue on relative alpha and theta power measures by cEEGrids

The findings of this study suggest that the effect of sleep restriction can be detected by an increase in alpha power in a selection of cEEGrids electrodes. The results showed that relative alpha power increased in five channels, indicating increased sleepiness. Previous studies have demonstrated that channels placed on the temporal lobe around the ear are sensitive to frequency bands relevant to fatigue [4, 57]. However, contrary to the hypothesis, the present study did not find an increase in theta band power. One possible explanation for this finding is that the level of fatigue experienced by the participants was relatively mild. While alpha power is typically increased during sleepiness, an increase in theta power only occurs after severe sleep deprivation and is considered an indicator of approaching sleep [25, 58, 59]. In the current study, the mean scores on the KSS did not exceed 7 (*M* = 6.14), indicating that participants were only moderately fatigued.

**Figure 7.**
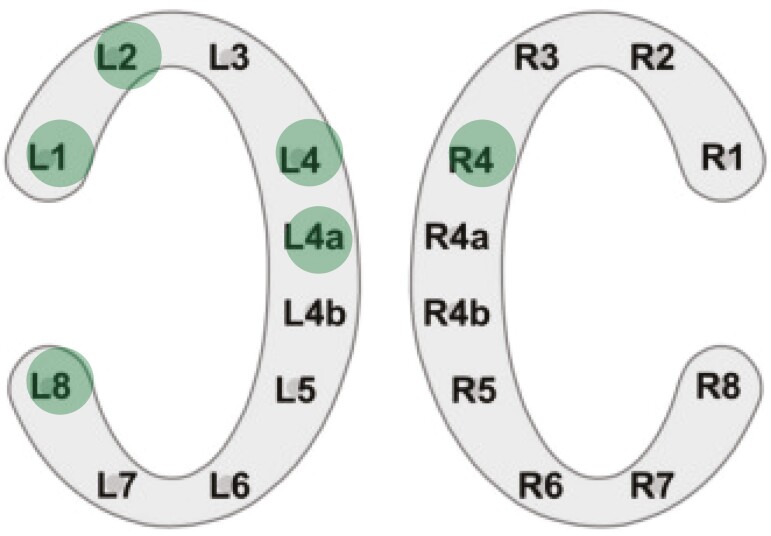
Selection of channels with significant differences in relative alpha band power after fatigue. Channels L01, L02, L04, L04a, and R04 revealed an increase after sleep restriction and relative alpha power of channels L01, L04, and L08 increased after a workday.

Interestingly, similar to past studies employing cEEGrids [39, 53], in the current study, a notable difference was found between electrode channels of the cEEGrids. Only five electrode channels, with just one located on the right array, exhibited a significant increase in relative alpha power after sleep restriction, while three channels demonstrated an increase only in the afternoon (Figure 7). Caffeine consumption might have influenced the results, as the study did not control for caffeine intake, and there was a significant increase in consumption after sleep restriction. Previous research indicates that both habitual caffeine consumption and acute caffeine intake are associated with a global reduction in alpha and theta power [60, 61]. Although no increase in theta power was observed, it is possible that this restricted the effect of alpha power. A further explanation for this inconsistency between channels is that certain channels may be more adept at detecting alpha power than others. For instance, Binias et al. [62] demonstrated that a decrease in alpha power in the left temporal lobe and the occipital area contributes to a faster reaction time, which could explain differences between the left and the right channels. Moreover, the lower channels, located in close proximity to the jaw muscles, tend to produce higher levels of noise due to muscle artifacts compared to the higher electrode channels. As a result, lower channels may produce weaker signals and are less sensitive, which could explain their inability to capture significant differences [63]. Furthermore, it is worth noting that cEEGrids are not positioned directly over the relevant brain regions, such as the parietal or frontal cortex, leading to weaker signals compared to common cap-EEG, with the exception of the temporal lobe [64]. This weaker signal can also be attributed to the shorter distance between the cEEGrids and the common reference electrodes, resulting in lower sensitivity [33, 53, 64].

To further address the differences between channels, correlations between electrode channels indicated that the alpha power of the channels were significantly related. This suggests that differences in detecting alpha power between channels can be explained by differences in sensitivity between electrode locations to alpha power after sleep restriction. Future devices could possibly require less electrodes, utilizing only the channels with high sensitivity. However, further research is needed to determine the significance of channel location and to identify optimal electrode configurations for capturing the most relevant fatigue-related signals using cEEGrids [64].

### The effect of fatigue on the KSS and the PVT

The KSS was utilized to determine subjective fatigue levels. The findings show that sleep restriction and prolonged time awake increased subjective sleepiness ratings, which is in line with previous studies [40, 44, 59, 65–67]. However, it is worth noting that the mean KSS scores in the current study did not surpass seven. Although participants woke up 3 hours earlier than their usual routine, they fell asleep earlier than their instructed bedtime, resulting in an average of 5 hours of sleep. Consequently, the level of sleep restriction was not high enough to result in severe fatigue.

In fatigue research, the KSS is commonly combined with the PVT. In the current study, the mean response speed of the PVT served as a measure of fatigue [18, 50]. Results showed that the mean response speed was reduced after sleep restriction, limited to the afternoon. This is in line with previous research showing that even minor sleep loss can negatively affect sustained attention [50, 68, 69]. Moreover, results suggest that prolonged time awake affects sustained attention at the end of duty, as seen in previous studies on early rising and its effects on alertness [65, 66]. However, no significant differences in sustained attention were observed between the first and second-morning tests, indicating that sleep restriction only affects sustained attention after prolonged time awake. A possible explanation for this is a study by Lamond and Dawson [70], in which performance on neurobehavioral tasks improved during the morning hours despite 24 hours of continuous wakefulness, attributed to the influence of the circadian rhythm.

To summarize, both the KSS and PVT outcomes showed a significant difference after sleep restriction. This effect is confirmed as a significant correlation was found between KSS scores and PVT performance. Although, previous studies have reported mixed results, with both significant and no correlations found [24, 25, 71, 72],

### The relationship between cEEGrids and traditional measures (KSS, PVT) of fatigue

To provide additional support for the evaluation of cEEGrids as a tool for detecting fatigue, a repeated-measures correlation analysis was conducted between the relative alpha band power of selected channels and the outcomes of the KSS and PVT. Results showed no significant correlations between the alpha power of the channels and the subjective KSS scores and the mean response speed of the PVT. This suggests that the differences in alpha power captured by means of the cEEGrids were not related to subjective sleepiness and objective alertness levels. This finding is inconsistent with previous research that has shown a strong association between relative alpha power measured by cap-EEG and subjective KSS scores following sleep restriction and have reported negative correlations between changes in alpha power and performance of vigilance tasks [24, 25, 73–75]. However, it has been demonstrated that sleepiness is best captured near the occipital lobe whereas cEEGrids are most sensitive to the temporal lobe [33, 73]. Moreover, the strongest changes of alpha power in vigilance tasks were found to be located at the frontal and parietal cortex [59, 74–76]. It is possible that the electrode placement of the cEEGrids are not a representative measure for obtaining subjective sleepiness or objective attention levels compared to cap-EEG [33]. Taken together with the fact that participants experienced only a mild degree of fatigue, this may have contributed to the observed outcomes. Furthermore, other studies have also reported a lack of significant correlation between alpha power and sustained attention following only mild or no sleep loss [73, 77]. These findings suggest that induction of severe sleep restriction may be necessary to observe correlations between vigilance and alpha power, which was not the case in the current study.

### Limitations

The current study has several limitations that should be taken into consideration when interpreting the results. Firstly, it is important to acknowledge that sleep regulation in the days leading up to the experiment was not controlled and the timing of tests did not align with individual peak fatigue levels dictated by circadian rhythm and chronotype due to restricted office hours [78]. Consequently, there were interindividual differences, as participants reported different sleepiness levels during the day following the sleep-restricted night. Moreover, the implemented earlier awakening time of 3 hours in the study did not effectively achieve the intended sleep restriction in all participants, failing to induce the intended level of fatigue, for which these variations may have constrained the observed effects. Nonetheless, the significant differences found in alpha power suggest that the cEEGrids are sensitive to the consequences of even mild sleep loss, emphasizing their potential utility in future fatigue studies. Secondly, although the relatively small sample size of 12 participants is not uncommon in within-participant EEG studies, it did affect the statistical power of a study [24, 34, 53, 79]. Moreover, it was decided not to apply counterbalancing of the order of the conditions, to mitigate potential after-effects of acute sleep restriction. In addition, a 1-week interval between measurements was implemented to alleviate the potential boredom effect [80, 81]. Nevertheless, it should still be acknowledged that the effect of the PVT might have been influenced by a boredom effect as a result of an order effect, potentially amplifying the difference in performance. While the PVT is known to have a small learning effect, the predetermined order of conditions could also have decreased the observed differences in the current design [82]. Finally, the use of a single-size adhesive of cEEGrids may have contributed to individual differences in signals, particularly for electrodes placed below the ear. It was observed that for some participants the adhesive was too large, causing the lower electrodes to approach the jaw muscles and possibly impacting signal quality. The acquisition of a reliable signal from the cEEGrid electrodes positioned above the ear proved challenging due to the presence of hair growth, which impeded optimal contact with the skin.

### Implications for future research/practice

The findings of this study support the hypothesis that cEEGrids can detect fatigue after both sleep restriction and extended time awake by capturing a difference in relative alpha power, although this distinction is not observed across all electrode channels. Further research is needed to enhance the sensitivity of all cEEGrids channels in detecting fatigue. For instance, by evaluating different electrode configurations and gaining a better understanding of the source locations associated with fatigue that can be accurately measured using cEEGrids [83]. Additionally, individual differences should be minimized by employing techniques such as forward modeling and creating fitted cEEgrids adhesives [64].

In practical field settings, such as cockpits or air traffic control operations rooms, a smaller set of electrodes suffices, offering advantages in terms of user convenience and mobility [3, 4]. However, despite its fast application, self-applying cEEGrids without training in a naturalistic setting can be challenging due to the necessary preparation steps, the use of gel, and limited adaptability of the sticker [84]. Therefore, improvements could include employing dry electrodes, lightweight portable recording equipment, or completely integrating EEG technology into headphones or headwear. Future research may focus on refining the cEEGrids for fatigue detection or comparing them with similar devices that already possess these enhancements in a strictly controlled setting before practical applications in the field.

Although current evaluation deems the use of PVT and KSS sufficient for field settings until developments are in place, this study shows the potential of cEEGrids in detecting fatigue after sleep restriction and extended time awake. By acknowledging the challenges associated with the practical implementation of the cEEGrids, the study provides suggestions for future research and development areas to refine the technology.

## Final Conclusions

The aim of this study was to investigate the use of cEEGrids as an objective and viable measure of fatigue for safety-critical industries, specifically focusing on aviation. The cEEGrids were able to capture differences in relative alpha power in a selection of electrode channels following both sleep restriction and a workday. These findings were partially consistent with outcomes of traditional fatigue measures. However, relative alpha power showed no correlations between subjective ratings of fatigue and response speed. Overall, this study provides insights into both the feasibility and limitations of utilizing cEEGrids as a means to measure fatigue. Even though further research is needed to fully explore the potential of cEEGrids or other ear-EEG devices in assessing fatigue after extreme sleep deprivation and investigating ways to optimize the quality of relevant EEG signals, the findings of this research lay the groundwork for future studies into the potential applications of ear-EEG as a fatigue detection tool.

## Notes

This paper is part of the *Sleep and Circadian Health in the Justice System* Collection.
